# Induction of regional chemokine expression in response to human umbilical cord blood cell infusion in the neonatal mouse ischemia-reperfusion brain injury model

**DOI:** 10.1371/journal.pone.0221111

**Published:** 2019-09-04

**Authors:** Nobuyasu Baba, Feifei Wang, Michiro Iizuka, Yuan Shen, Tatsuyuki Yamashita, Kimiko Takaishi, Emi Tsuru, Sachio Matsushima, Mitsuhiko Miyamura, Mikiya Fujieda, Masayuki Tsuda, Yusuke Sagara, Nagamasa Maeda

**Affiliations:** 1 Center for Innovative and Translational Medicine, Kochi Medical School, Kochi University, Kochi, Japan; 2 Department of Pharmacy, Kochi Medical School Hospital, Kochi, Japan; 3 Institute for Laboratory Animal Research, Science Research Center, Kochi University, Kochi, Japan; 4 Department of Obstetrics and Gynecology, Kochi Medical School, Kochi University, Kochi, Japan; 5 Department of Pediatrics, Kochi Medical School, Kochi University, Kochi, Japan; Universita degli Studi di Napoli Federico II, ITALY

## Abstract

Regenerative medicine using umbilical cord blood (UCB) cells shows promise for the treatment of cerebral palsy. Although the efficacy of this therapy has been seen in the clinic, the mechanisms by which UCB cells interact and aid in the improvement of symptoms are not clear. We explored the chemokine expression profile in damaged brain tissue in the neonatal mouse ischemia-reperfusion (IR) brain injury model that was infused with human UCB (hUCB) cells. IR brain injury was induced in 9-day-old NOD/SCID mice. hUCB cells were administered 3 weeks post brain injury. Chemokine expression profiles in the brain extract were determined at various time points. Inflammatory chemokines such as CCL1, CCL17, and CXCL12 were transiently upregulated by 24 hours post brain injury. Upregulation of other chemokines, including CCL5, CCL9, and CXCL1 were prolonged up to 3 weeks post brain injury, but most chemokines dissipated over time. There were marked increases in levels of CCL2, CCL12, CCL20, and CX3CL1 in response to hUCB cell treatment, which might be related to the new recruitment and differentiation of neural stem cells, leading to the induction of tissue regeneration. We propose that the chemokine expression profile in the brain shifted from responding to tissue damage to inducing tissue regeneration. hUCB cell administration further enhanced the production of chemokines, and chemokine networks may play an active role in tissue regeneration in neonatal hypoxic-ischemic brain injury.

## Introduction

Cerebral palsy (CP) is a permanent movement disorder caused by abnormal development or damage to brain tissue during embryonic development and postnatally. The prevalence of CP is 2 to 3 per 1000 live births. No curative therapy for CP is available; thus, patients with CP can only undergo symptomatic treatments, such as rehabilitation [1]. Potential treatments in the field of regenerative medicine are under development. A clinical trial of autologous or allogenic umbilical cord blood (UBC) cell transfusions to children with CP showed promising efficacy in improving brain connectivity and gross motor function [2,3]. However, more research is required to determine the safety, efficacy, and mechanism of action of this treatment.

UCB has been used safely for the treatment of aplastic anemia and certain types of leukemia for many years. Recently, UCB cells, including hematopoietic and mesenchymal stem cells, as well as other types of progenitor cells, have been considered potential sources of cell therapy to treat neurodegenerative disorders, cardiovascular diseases, and certain tumor types [4]. Evidence suggests that UCB cells act via paracrine signaling on endogenous cells to facilitate tissue repair or improvement of function [5].

Chemokines are cell-to-cell signaling molecules that lead to cell migration, survival, or activation in healthy steady-state conditions and in inflammation [6–8]. It is well known that chemokines play an active role in bringing stem cells to the site of inflammation [9]. In addition, chemokines have been shown to act on stem cells or damaged central nerve systems to either worsen damage or induce tissue repair [10–13]. Considering these pleiotropic functions of chemokines, it is possible that chemokines could exert therapeutic effects in treatments using UCB. UCB cells might interact with the cells in lesion area and could induce chemokine production by other cells.

We used the neonatal mouse ischemia-reperfusion (IR) brain injury model to examine chemokine expression profiles in the regionally damaged brain after tissue injury as well as after human UCB (hUCB) cell transfusion. We found that there were unique chemokine expression patterns in accordance with duration of time after brain injury (from 24 hours to 5 weeks post injury), and hUCB cells induced chemokine expression in damaged tissue.

## Materials and methods

### Mice and neonatal ischemia-reperfusion brain injury model

NOD/SCID (NOD.CB17-Prkdcscid/J) mice (Charles River Laboratories, Kanazawa, Japan) were used in this study. Neonatal ischemia-reperfusion (IR) brain injury was induced as previously described [14]. In brief, 9-day postnatal NOD/SCID mice were anesthetized with 2% isoflurane. The pups were placed on the heating pad for control of body temperature throughout the surgery until they recover from anesthesia. The right common carotid artery was occluded using an aneurysm clip (Mizuho, Tokyo, Japan). The pups were placed in a hypoxia chamber maintained with 8% oxygen for 120 minutes. Reperfusion was performed by unclamping the artery and exposing the pups to normoxic conditions. Pups were returned to their dams after these manipulations. Mice were kept in specific-pathogen-free condition with food and water *ad libitum*, 12 hours of light-dark cycle and clean air ventilation at controlled temperature and humidity, according to animal welfare practices.

**Fig 1:**
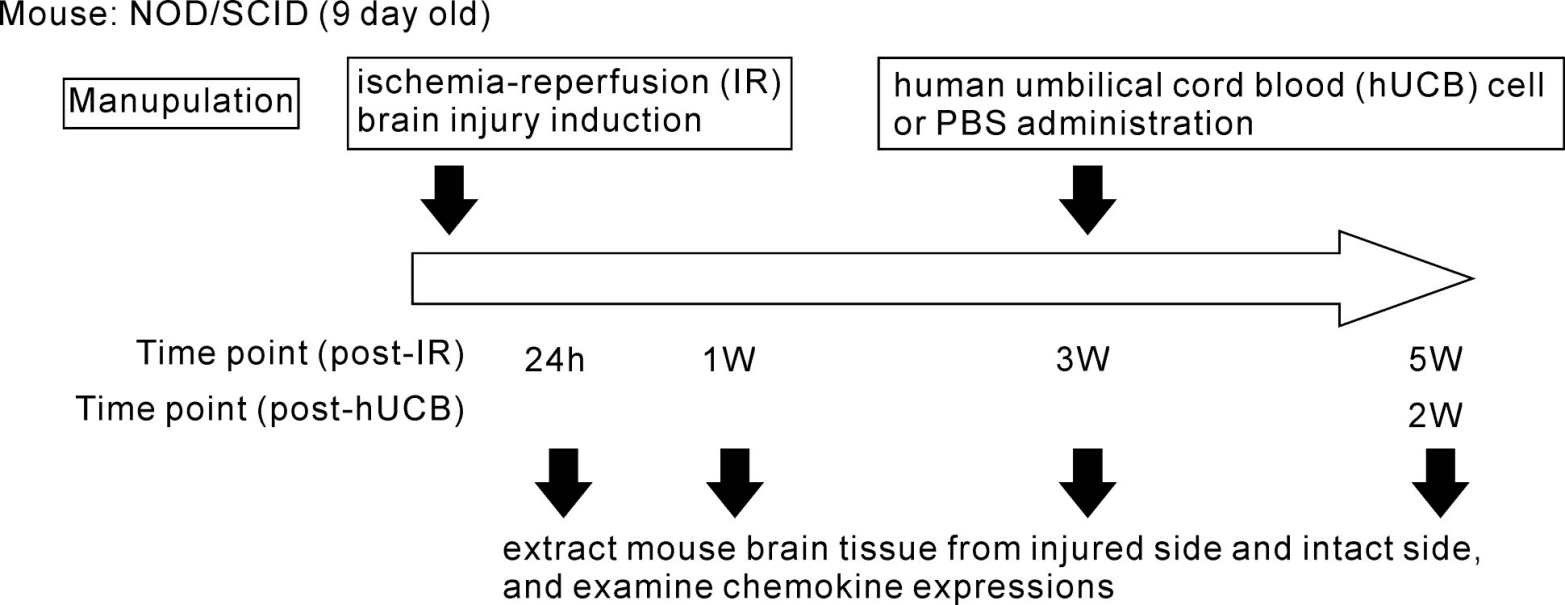
Schematic illustration of experimental procedure.

Fig 1 shows the schema of the experimental procedure. At each check point, IR brain injury mice (n = 3–5 for each points) were sacrificed by either overexposure of isoflurane inhalation anesthesia (for neonates) or cervical spine fracture dislocation (for adult mice). Brain tissues were collected and sampled from both the injured (right) side and the contralesional intact (left) side from individual mice. In some experiments, healthy mice (non-IR injury) were used as reference controls.

We performed MRI scanning to see the severity of brain tissue damage in some of experiments as our group previously reported [14]. Also at the time of craniotomy, we confirmed the injury by macroscopic observation for all mice used. The samples of mouse brains which have same injury area or degree were assigned to use for the experiments.

### Human umbilical cord blood (hUCB) cell transfusion

Mice (n = 5) with IR brain injury were transfused with mononuclear cells prepared from hUCB (provided by the RIKEN BRC through the Project for Realization of Regenerative Medicine and the National Bio-Resource Project of the MEXT Japan). At 3 weeks post-IR injury, mice were injected intravenously by the tail vein with 5 × 10^6^ hUCB cells in 100 μL of phosphate buffered saline (PBS). Untreated control mice (n = 5) were injected with the same amount of PBS.

Experiments were performed under the approval of the institutional animal experiment committee at Kochi University, Kochi, Japan (approval number L-00037), and the use of human materials was under agreement with RIKEN BRC (MTA number CM00069) and certified with a local research ethics committee at Kochi Medical School, Kochi University (approval number 21–49).

We determined the sample size according to our preceding study [14] where we could see the significant differences in some chemokine secretions by n = 4. We used the Antibody Array methods for profiling a large number of cytokine/chemokine expression simultaneously in this study. Since this method was considered as less sensitive, we increased the sample size up to 5 to evaluate the tendency of chemokine production.

### Chemokine determination and profiling

Tissue lysate was prepared by homogenization in RIPA Lysis and Extraction Buffer (Thermo Fisher Scientific, Waltham, MA, USA) in the presence of a complete cocktail of protease inhibitors (Roche, Basel, Switzerland). Tissue debris was removed by centrifugation at 1000 × g for 10 minutes. Total protein concentrations in the tissue lysate were quantified by a BCA Protein Assay Kit (Thermo Fisher Scientific).

Humoral factor expressions, including chemokines, in the brain tissue lysate were determined by an antibody-array method. RIPA-extracted samples containing 1 mg of total proteins were applied onto a Mouse Cytokine Antibody Array C3 (RayBiotec, Norcross, GA, USA) and developed per the manufacturer’s instructions. Signals were acquired and digitalized by LAS4000miniEPUV (Fuji Film, Tokyo, Japan) and analyzed by ImageJ software (National Institutes of Health, Bethesda, MD, USA). Data were normalized with controlled positive and negative spots provided in the Ab array membrane to enable relative sample-to-sample comparisons.

Some selected chemokines were further quantified by bead array methods, using the following reagent kits; LegendPlex (from BioLegend, San Diego, CA, USA) for CCL3, CCL4, CCL11, CCL17, CXCL10, and CXCL13; HQPLEX (from Spherotech, Lake Forest, IL, USA) for CCL9, CCL12, and CXCL2 and Firefly (from Abcam Cambridge, UK) for CCL24, CCL25, and CXCL4. The bead-array experiments were performed per each manufacturer’s instructions, and data were acquired using BD LSRFortessa (BD Biosciences, San Jose, CA, USA) and analyzed by software provided by the bead supplier or FCAP Array (Soft Flow, Pecs, Hungary).

### Immunohistologic evaluation of brain tissue in mice

At 1 week after IR injury (control group) and 24 hours (for hUCB cell tracking) or 2 weeks after hUCB cell administration (hUCB-treatment group), brains were collected and fixed with 4% paraformaldehyde. Coronal brain sections were obtained using a cryostat (CM3050S; Leica, Bensheim, Germany) at the thickness of 10 μm slices. Immunostaining for chemokines and cellular markers was mostly performed as previously described [15,16]. In brief, frozen sections were blocked with 20% Block Ace (DS Pharma Biomedical, Osaka, Japan) in 0.1 M phosphate buffer (PB) containing 0.01% saponin (Nacalai Tesque, Kyoto, Japan), followed by incubation with a cocktail of primary antibodies against CCL9, CXCL12, CXCL9, CXCL1, CX3CL1, or CXCL16 (Abcam) and anti-NeuN, anti-Iba1 (Merck, Kenilworth, NJ, USA) anti-GFAP (BD Biosciences), anti-O4 (R&D systems, Minneapolis, MN, USA) or anti-HLA-ABC (Thermo Fisher Scientific) antibodies in dilution buffer (0.1 M PB containing 5% Block Ace and 0.01% saponin) for 1 day at 4°C. After incubation with the primary antibody, the sections were washed and incubated with fluorescently labeled secondary antibodies; DyLight 488 anti-rabbit IgG (BioLegend), Alexa-Fluor 488 anti-rat IgG (Cell Signaling Technology, Danvers, MA, USA) and Alexa-Fluor 594 anti-mouse IgM or IgG (Thermo Fisher Scientific) in dilution buffer for 1 day at 4°C. Samples were covered with the VECTASHIELD mounting medium with DAPI (Vector Laboratories, Burlingame, CA, USA) and analyzed using a fluorescence microscope (BZ-9000; Keyence, Osaka, Japan). Microscopic analysis was performed at high-magnification for three randomly selected area of intact side or injure side of brain and evaluated the human antigen expressing cells or co-expression of chemokines and cellular markers.

### Statistical analysis

Data are depicted as mean ± SEM. Kolmogorov–Smirnov test or Student's t test was used to compare findings between groups using the Prism 6 software (GraphPad Software, San Diego, CA, USA). P values < 0.05 were considered significant.

## Results

### Ischemia-reperfusion injury enhanced chemokine production in injured side brain tissue

**Fig 2:**
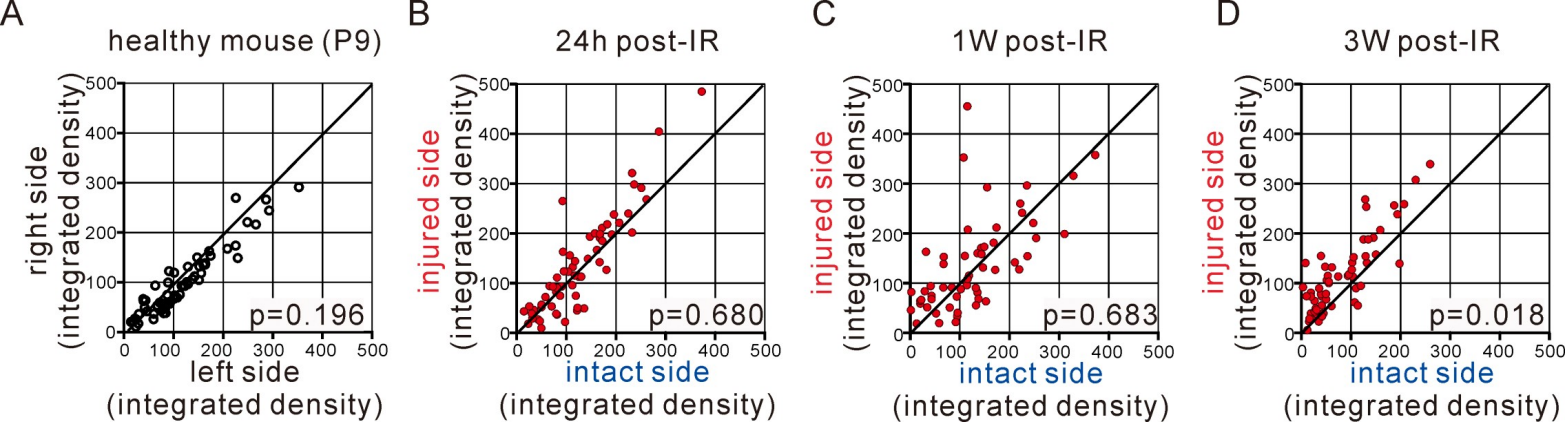
Ischemia-reperfusion (IR) brain damage promoted cytokine/chemokine expression in the injured side. **(A)** Expression levels in healthy mice (P9, right vs left side brain) are shown as reference controls. **(B-D)** Plots are depicted as expression levels in the intact side on the x axis and in the injured side on the y axis after 24 hours (B), 1 week (C), and 3 weeks (D) post-IR. Data are the mean of expression levels in tissue lysate detected as integrated density. The p values comparing between injured side and intact side were indicated. P9, 9 days postnatal.

As shown in Fig 2, about 60 molecules, including chemokines, were detected and up-regulated in the injured side of the brain compared with the intact side. These up-regulations were observed as soon as 24 hours post-IR injury and prolonged for up to 1 to 3 weeks after induction of injury. These findings indicate that in the injured brain, many humoral factors were seen in response to tissue damage; this reaction may have induced tissue damage or repair. The significant induction of cytokines and chemokines was observed at 3 weeks after injury, although there was a gradual decrease in some levels.

**Fig 3:**
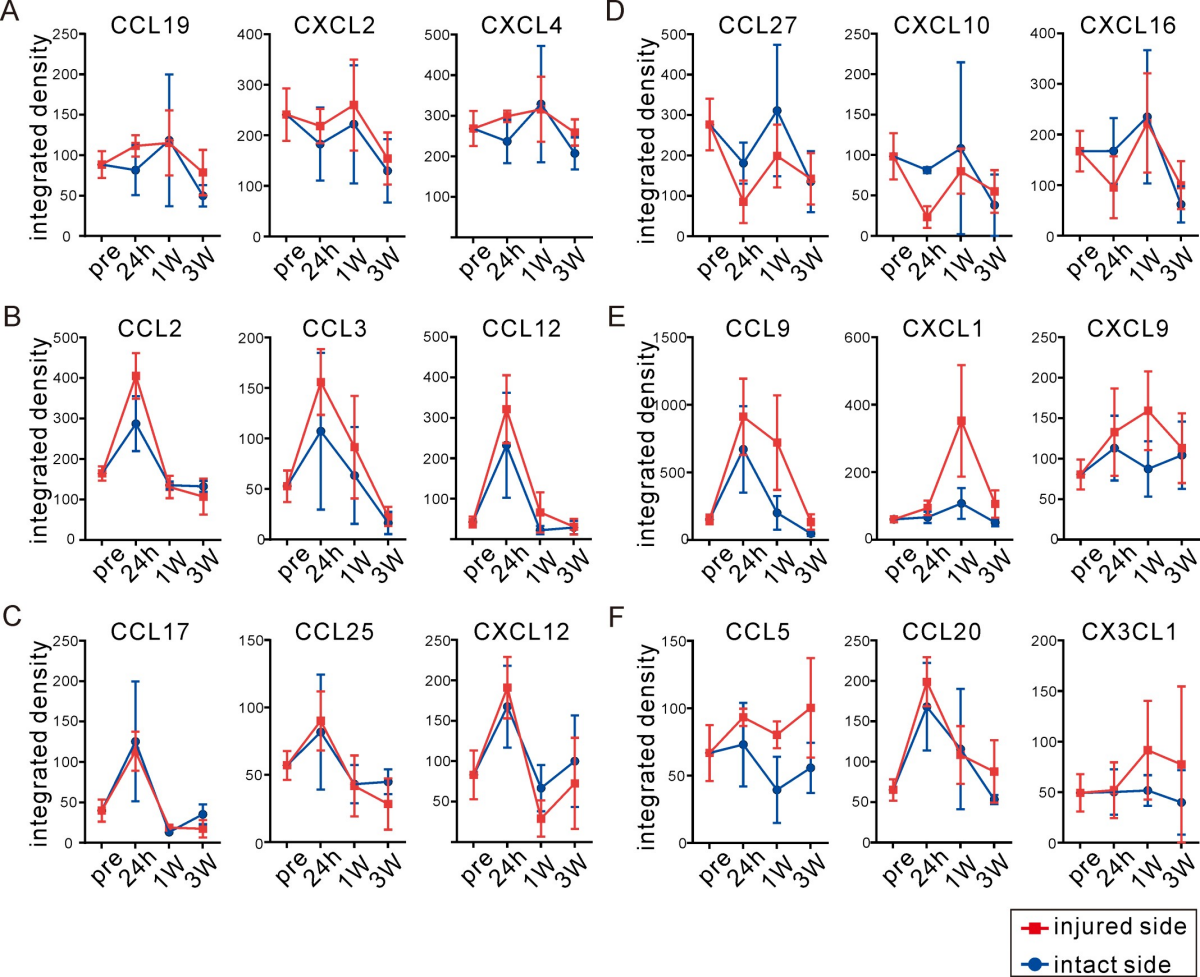
Chemokine promoted by ischemia-reperfusion (IR) brain damage showed unique production patterns. **(A-F)** Chemokines were divided into six groups according to individual patterns. Chemokines not affected by IR injury (A); chemokines transiently up-regulated at 24 hours post-IR on the injured side (B) or on both sides (C); chemokines slightly inhibited by IR injury (D); chemokines with higher expression at 1 week post-IR (E) and prolonged expression until 3 weeks post-IR (F). Integrated density data detected by antibody array are shown as mean ± SEM. Time course determined is indicated as pre (before IR in P9 intact mice), 24 hours, 1 week and 3 weeks Blue circles indicate the intact side and red squares indicate the injured side.

We found different expression patterns for different chemokines. CCL19, CXCL2, and CXCL4 did not change with tissue damage (Fig 3A). Other chemokines, including CCL2, CCL3, and CCL12, were transiently up-regulated in the injured side at 24 hours post-IR injury and decreased over time (Fig 3B). CCL17, CCL25, and CXCL12 were up-regulated in both the intact and injured sides of the brain (Fig 3C). CCL27, CXCL10, and CXCL16 were inhibited by tissue damage and restored in the injured side as time progressed (Fig 3D). CCL9, CXCL1, and CXCL9 reached peak expression levels at 1 week post-IR injury, a finding distinct from other chemokines (Fig 3E). CCL5, CCL20, and CX3CL1 showed prolonged expression by 3 weeks post-IR injury (Fig 3F). These findings might indicate that each chemokine has its own expression pattern in response to tissue damage. Different chemokines may contribute to tissue damage or initiate tissue repair. In addition, it is possible that desired responses regarding tissue maintenance or repair could not be sustained, as most up-regulated chemokines decreased over time.

### hUCB cell transfusion re-induced chemokine production in injured tissue

**Fig 4:**
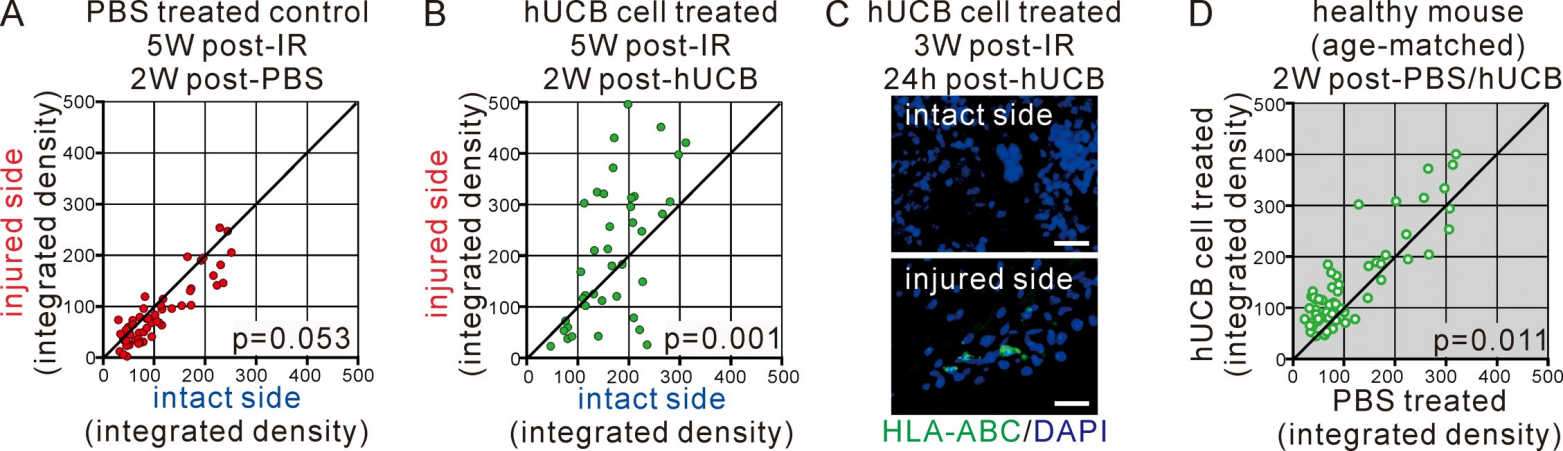
Human UCB (hUCB) cells were localized and re-enhanced cytokine/chemokine expression in the injured side. **(A, B)** Cytokine and chemokine expressions were determined at 5 weeks post-IR without treatment (PBS treated control) (A) or with hUCB cell treatment at 3 weeks post-IR (B). Data are shown as in Fig 2. The p values comparing between injured side and intact side were indicated. **(C)** Brain tissue sections at 24 hours after hUCB cell treatment for 3 weeks post-IR mice were immunohistochemically stained for human antigen HLA-ABC, visualized in green. Counterstaining with DAPI (in blue) was also performed. Merged images of the intact side and injured side of brain tissue were shown. Bars = 20 μm. Data are one of representative observations. **(D)** The effect of hUCB cell treatment of healthy mice was compared to un-treated (PBS) age- and treatment timing-matched mice as reference controls. Data are shown as mean of expression levels. The p value comparing between PBS treated and hUCB cell treated was indicated.

As shown in Fig 4A, at 5 weeks post-IR injury (PBS treated control), most humoral factors stopped being produced and no differences were noticed between the injured and intact sides of the brain. Mice treated with hUCB cells showed highly significant increases in humoral factors in the injured side of the brain (Fig 4B). We confirmed by immunohistological assessment that hUCB migrated to the site of damaged brain tissue selectively, but not on the intact side of the brain (Fig 4C). Healthy mice showed sifnificant but only a limited reaction compared to IR injury mice following injection of hUCB cells (Fig 4D). These data demonstrated that both tissue damage and hUCB transfusion may represent specific factors that induce humoral molecule secretions specifically on the injured side.

**Fig 5:**
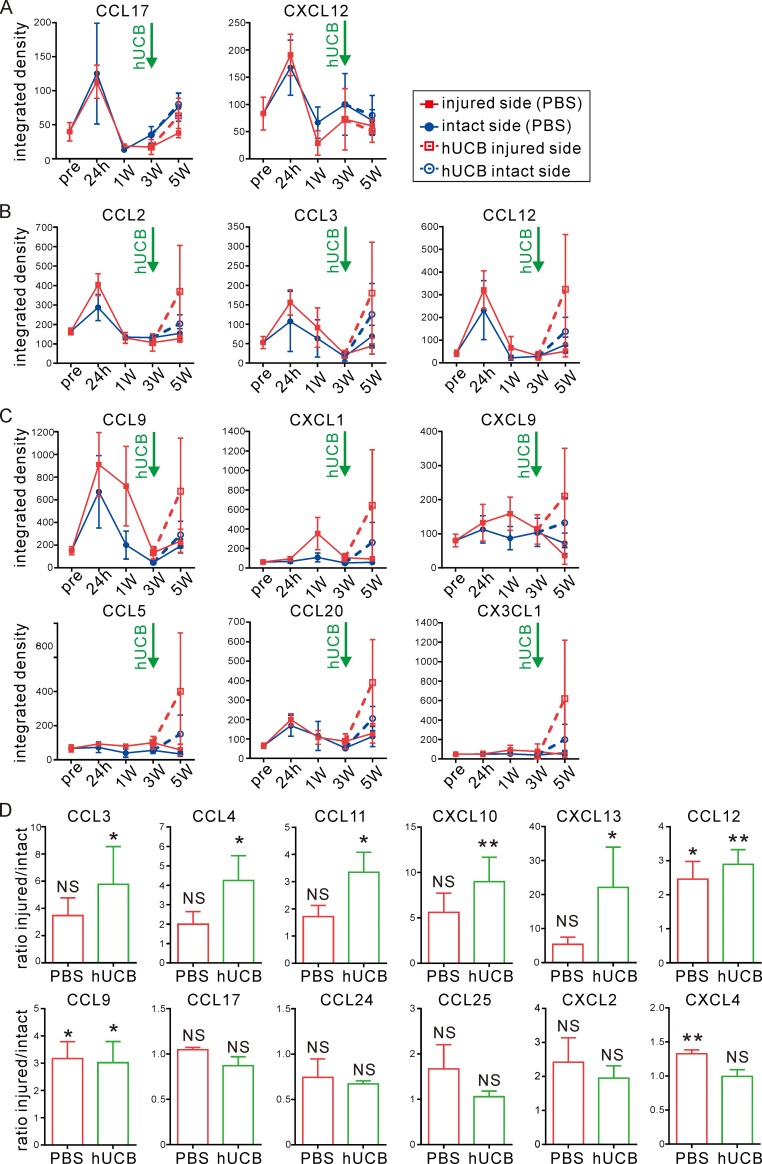
Human UCB (hUCB) cells enhanced chemokine production in the injured side. **(A-C)** Expression patterns of chemokines after hUCB cell treatment at 3 weeks post-IR were determined. Time course determined is indicated as pre (before IR in P9 intact mice), 24 hours, 1 week, 3 weeks, and 5 weeks (2 weeks post-hUCB). According to the expression pattern, three groups were found; hUCB cells had no effect (A), restored degraded chemokines (B), or further enhanced chemokines slightly changed by IR in the injured side (C). Data are shown as mean ± SEM as explained in Fig 3. Dashed and open symbols indicate the hUCB cell treatment group. **(D)** Ratio of injured / intact (fold induction of expression level on injured side compared to intact side) at 5 weeks post-IR with or without hUCB cell treatment. Data are mean ± SEM, quantified by the bead-based quantitative assay. *: p<0.05, **: P<0.01, NS: not significant.

In the analysis of precise chemokine expression patterns, we found that neither CCL17 nor CXCL12 responded to damage or hUCB cell transfusion (Fig 5A). CCL2, CCL3, and CCL12 were re-upregulated by hUCB cell transfusion (Fig 5B). Chemokines that showed delayed expression peaks at 1 week post-IR injury (CCL9, CXCL1, and CXCL9) or prolonged expression up to 3 weeks post-IR injury (CCL5, CCL20, and CX3CL1) were greatly increased in the injured side after hUCB cell transfusion (Fig 5C). For selected chemokines, we confirmed a significant induction induced by hUCB cell administration by the bead-based quantitative assay. As shown in Fig 5D, for chemokines CCL3, CCL4, CCL11, CXCL10, and CXCL13, the ratio of injured / intact chemokine expression was significantly increased by hUCB cell treatment. CCL12 and CCL9 were up-regulated for both the control and treated groups. Other chemokines showed no differences in expression between treated and control mice, although CXCL4 was significantly up-regulated in the PBS group (at 5 weeks post-IR injury), whereas hUCB cells showed no effects. These expression patterns might help in repairing damaged tissue and supporting recovery of function by recruiting and/or inducing differentiation of the intrinsic neural stem cell population around damaged tissue.

### Residential cells composing brain tissue were the source of chemokines

**Fig 6:**
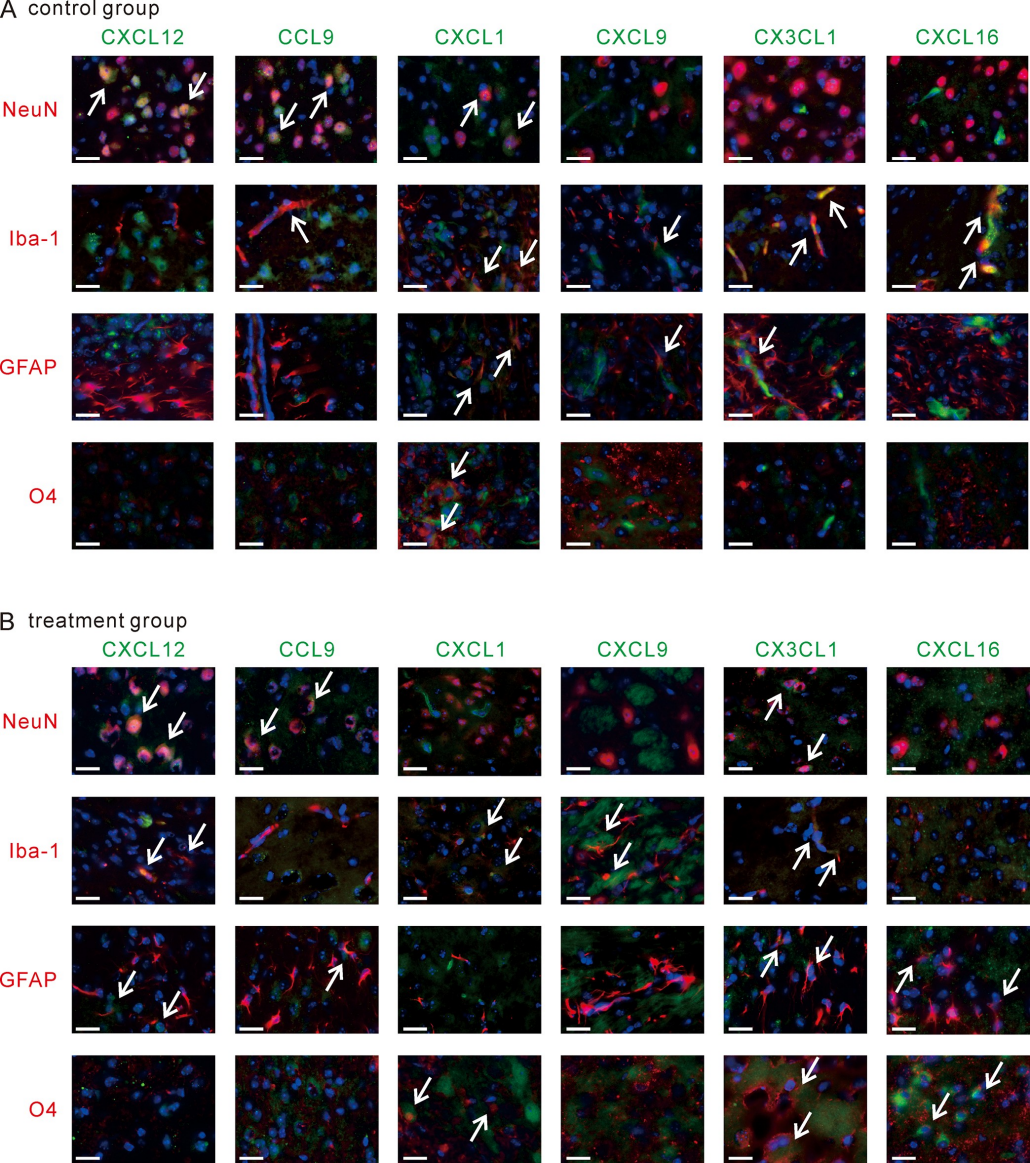
Brain composing cells were the source of chemokines. **(A, B)** Brain tissue sections at 1 week post-IR in the control group (A) or 5 weeks post-IR in the hUCB cell treatment group (B) were immunohistochemically stained for chemokines and brain cellular markers. Chemokines are shown in green, and cell markers are shown in red. Counterstaining with DAPI (in blue) was also performed, and merged images are shown to allow visualization of co-expressed chemokines and cell markers. Arrows indicate co-expression signals. Bars = 20 μm. Data are one of representative observations.

As shown in Fig 6A, we found different and unique cellular sources according to the type of chemokines. NeuN-positive mature neurons produced CXCL12 in both the hUCB cell treatment group and PBS injected control group. CCL9 was induced by NeuN-positive and Iba1-positive microglias at 1 week post-IR injury. CXCL9 and CX3CL1 were produced by Iba1-positive cells and GFAP-positive astrocytes, but not by mature neurons. CXCL1 was produced by a number of different cells, including neurons, microglias, astrocytes, and O4-expressing oligodendrocytes. CXCL16, suppressed immediately after injury, was secreted only by microglias.

Re-enhancement of chemokine secretion induced by hUCB cell transfusion partially altered the cellular source of chemokines (Fig 6B). For instance, CXCL12 was secreted by not only neurons but also microglias and astrocytes after hUCB cell transfusion. CX3CL1 was produced by neurons and oligodendrocytes in response to tissue damage, and hUCB cells also initiated CX3CL1 secretion by microglias and astrocytes. CCL9 from neurons and CXCL9 from microglias were sustained by hUCB cell transfusion, but neurons and astrocytes stopped secreting CXCL1 after hUCB cell transfusion. CXCL16 production was switched from microglias after tissue injury to astrocytes and oligodendrocytes upon hUCB cell transfusion.

These findings demonstrate that different cells in brain tissue can be the source of chemokines, and these cells respond to either tissue damage or hUCB cell injection. This might subsequently create a pathologic condition and/or facilitate tissue repair. The functions of these chemokines and the mechanisms of how tissue damage or hUCB cell injection acts on brain cells and induces these chemokines need to be further elucidated.

## Discussion

We showed that damage to brain tissue induced changes in local chemokine expression patterns, and hUCB cell injection altered some of these patterns. We confirmed that there are unique expression patterns in accordance with the time since initial brain injury, which is in agreement with a previous study using a brain injury model in rats that showed the induction of some cytokines and chemokines in response to tissue damage [17]. For the first time in the scientific literature, we demonstrated that there was a strong induction of chemokine secretions, specifically to damaged brain tissue, in response to hUCB cell injections, thus supporting the efficacy of this treatment in an animal model of brain injury.

It was well known that tissue damage induces inflammatory conditions, and that inflammation might induce further tissue damage. At the same time, this inflammation is necessary for tissue repair or regeneration [12,18]. In this study, once tissue damage occurred, most chemokines were upregulated within 24 hours of tissue damage. However, most of those increases were transient and decreased over time. A few chemokines were up-regulated after a few weeks. Although these findings were detected feeble differences by the Antibody Array method, it was trustworthy since a part of our data were confirmed its significance by the quantitative analysis in our preceding study [14]. Those molecules could possibly signal damaged tissue to initiate tissue repair. However, the fact that these reactions were not prolonged as well as decreased over time, may explain why patients with CP do not show spontaneous recovery or improvements.

We also demonstrated that hUCB cell injections triggered the production of chemokines that were released as an acute reaction against tissue damage but decreased over time. Those chemokines might activate tissue repair or protect the nervous system. Indeed, CCL5 and CX3CL1 have been shown to play an active role in neuronal cell survival and protection [19–22]. Other chemokines, such as CCL2 and CXCL12, are known to be involved with stem cell recruitment or differentiation in cases of ischemic tissue damage [23,24]. We previously demonstrated that CCL11 could activate the proliferation of intrinsic neural stem cells from the subventricular zone and induce the migration of these cells to the site of damaged tissue [14]. The chemokine dynamics that occur in response to both damaged tissue and hUCB cell transfusion might be the key to tissue repair or regeneration.

We also found that CCL2 or CXCL1 was upregulated by hUCB cell injection. These are typical inflammatory chemokines [25,26]. We used the NOD/SCID mice in this study to avoid rejection and increase the chance of human cell engrafts in mice. We confirmed that there were sifnificant but only limited differences between healthy mice injected with hUCB cells and PBS treated healthy mice in cytokine/chemokine expression profile in our experimental settings (Fig 4D). It is possible that hUCB cell injections induce undesired inflammation around damaged tissue initiated by residual immune cells in the immunodeficient mice. The function and role of chemokines require further investigation to clarify their balance with respect to tissue damage and repair.

Brain cells such as neurons and glial cells are capable of secreting chemokines [27–29]. We confirmed that, in tissue from the damaged side of the brain, brain cells were secreting unique expression patterns of chemokines. An acute (24 hours post-IR injury) chemokine such as CXCL12 was produced by NeuN-positive neurons, and CCL9 or CXCL9 which were grouped the semi-acute or chronic type of chemokines (1 week to 3 weeks post-IR injury) were secreted by microglias and astrocytes. Once mice were treated with hUCB cells, we saw re-activation of chemokine secretion in the injured side of the brain. hUCB cells were attracted to the tissue injury, and they might stimulate brain cells to become stronger chemokine producers. Our data demonstrated that after hUCB cell administration, some brain cells acquired the capacity to express a chemokine that was not seen only in tissue injury (Fig 6). hUCB cell treatment might lead to the activation of chemokine networks at the site of tissue injury, which may promote neuronal protection, differentiation, and proliferation to assist with tissue self-repair and/or regeneration.

A mouse model of IR brain damage was used in this study to investigate the effect of treatment using hUCB cells at the mouse age of approximately 4 weeks old. This is the murine age of weaning, which fits the age of the children treated in the clinical study [2]. The chemokine expression dynamics, as reported here in response to tissue damage and hUCB cell treatment, would be investigated in CP patients and/or patients with other types of disease, and other animal disease models. Moreover, how those chemokines function in tissue repair or regeneration and the relevance of this regulation to therapeutic efficacy are important issues for future research.

UCB cells are a remarkable new strategy that can be used in regenerative medicine. The positive efficacy of UCB treatment was reported, and a clinical trial using UCB to treat CP has been reported worldwide [2–4,30]. It is important to confirm the safety of this treatment and provide scientific knowledge regarding the mechanisms behind this treatment. In the present animal study, we provided evidence that chemokines are secreted in damaged brain tissue in response to both injured signals and hUCB cell treatment. These findings may help support the efficacy of this treatment in humans, although additional studies to support these findings are needed.
